# Feasibility of Screening for Chromosome 15 Imprinting Disorders in 16 579 Newborns by Using a Novel Genomic Workflow

**DOI:** 10.1001/jamanetworkopen.2021.41911

**Published:** 2022-01-04

**Authors:** David E. Godler, Ling Ling, Dinusha Gamage, Emma K. Baker, Minh Bui, Michael J. Field, Carolyn Rogers, Merlin G. Butler, Alessandra Murgia, Emanuela Leonardi, Roberta Polli, Charles E. Schwartz, Cindy D. Skinner, Angelica M. Alliende, Lorena Santa Maria, James Pitt, Ronda Greaves, David Francis, Ralph Oertel, Min Wang, Cas Simons, David J. Amor

**Affiliations:** 1 Diagnosis and Development, Murdoch Children’s Research Institute, Royal Children’s Hospital, Melbourne, Parkville, Victoria, Australia; 2 Faculty of Medicine, Dentistry and Health Sciences, Department of Paediatrics, University of Melbourne, Parkville, Victoria, Australia; 3 E.D.G. Innovations and Consulting, St Kilda, Victoria, Australia; 4 Centre for Epidemiology and Biostatistics, Melbourne School of Population and Global Health, University of Melbourne, Carlton, Victoria, Australia; 5 Genetics of Learning Disability Service, Hunter Genetics, Waratah, New South Wales, Australia; 6 Departments of Psychiatry, Behavioral Sciences and Pediatrics, University of Kansas Medical Centre, Kansas City, Kansas; 7 Molecular Genetics of Neurodevelopment, Department of Women's and Children's Health, University of Padua, Padua, Italy; 8 Istituto di Ricerca Pediatrica (IRP), Città della Speranza, Padua, Italy; 9 Center for Molecular Studies, J.C. Self Research Institute of Human Genetics, Greenwood Genetic Center, Greenwood, South Carolina; 10 Cytogenetics and Molecular Laboratory, Institute of Nutrition and Food Technology, University of Chile, Santiago, Chile; 11 Centre for Diagnosis and Treatment of Fragile X Syndrome (CDTSXF), INTA University of Chile, Santiago, Chile; 12 Victorian Clinical Genetics Services, Murdoch Children’s Research Institute, Royal Children’s Hospital, Melbourne, Victoria, Australia; 13 Translational Bioinformatics, Murdoch Children’s Research Institute, Royal Children’s Hospital, Melbourne, Victoria, Australia; 14 Neurodisability and Rehabilitation, Murdoch Children’s Research Institute, Royal Children’s Hospital, Melbourne, Victoria, Australia

## Abstract

**Importance:**

Newborn screening for Angelman syndrome (AS), Prader-Willi syndrome (PWS), and chromosome 15 duplication syndrome (Dup15q) may lead to benefit from early diagnosis and treatment.

**Objective:**

To examine the feasibility of newborn screening for these chromosome 15 imprinting disorders at population scale.

**Design, Setting, and Participants:**

In this diagnostic study, the validation data set for the first-tier *SNRPN* test, called methylation-specific quantitative melt analysis (MS-QMA), included 109 PWS, 48 AS, 9 Dup15q, and 1190 population control newborn blood spots (NBS) and peripheral tissue samples from participants recruited from January 2000 to December 2016. The test data set included NBS samples from 16 579 infants born in 2011. Infants with an NBS identified as positive for PWS, AS, or Dup15q by the first-tier test were referred for droplet digital polymerase chain reaction, real-time polymerase chain reaction, and low-coverage whole-genome sequencing for confirmatory testing. Data analyses were conducted between February 12, 2015, and August 15, 2020.

**Results:**

In the validation data set, the median age for the 77 patients with PWS was 3.00 years (IQR, 0.01-44.50 years); for the 46 patients with AS, 2.76 years (IQR, 0.028 to 49.00 years); and for the 9 patients with Dup15q, 4.00 years (IQR, 1.00 to 28.00 years). Thirty-eight patients (51.4%) in the PWS group, 20 patients (45.5%) in the AS group, and 6 patients (66.7%) in the Dup15q group who had sex reported were male. The validation data set showed MS-QMA sensitivity of 99.0% for PWS, 93.8% for AS, and 77.8% for Dup15q; specificity of 100% for PWS, AS, and Dup15q; positive predictive and negative predictive values of 100% for PWS and AS; and a positive predictive value of 87.5% and negative predictive value of 100% for Dup15q. In the test data set of NBS samples from 16 579 infants, 92 had a positive test result using a methylation ratio cut-off of 3 standard deviations from the mean. Of these patients, 2 were confirmed to have PWS; 2, AS; and 1, maternal Dup15q. With the use of more conservative PWS- and AS-specific thresholds for positive calls from the validation data set, 9 positive NBS results were identified by MS-QMA in this cohort. The 2 PWS and 2 AS calls were confirmed by second-tier testing, but the 1 Dup15q case was not confirmed. Together, these results provided prevalence estimates of 1 in 8290 for both AS and PWS and 1 in 16 579 for maternal Dup15q, with positive predictive values for first-tier testing at 67.0% for AS, 33.0% for PWS, and 44.0% for combined detection of chromosome 15 imprinting disorders for the validation data set.

**Conclusions and Relevance:**

The findings of this diagnostic study suggest that it is feasible to screen for all chromosome 15 imprinting disorders using *SNRPN* methylation analysis, with 5 individuals identified with these disorders out of 16 579 infants screened.

## Key Points

**Question:**

Is newborn screening feasible for chromosome 15 imprinting disorders, including Prader-Willi, Angelman, and Dup15q syndromes, using *SNRPN* methylation analysis?

**Findings:**

This diagnostic study involved validation of a novel methylation test on 1356 samples, showing high sensitivity and specificity and positive and negative predictive values to differentiate newborn blood spots and blood, saliva, and buccal DNA of 109 Prader-Willi, 48 Angelman, and 9 Dup15q patient samples from neurotypical control samples. Newborn blood spots from 16 579 infants from the general population were then tested, identifying 2 with Prader-Willi syndrome, 2 with Angelman syndrome, and 1 with Dup15q syndrome.

**Meaning:**

The findings of this study suggest that it is feasible to screen for all chromosome 15 imprinting disorders using *SNRPN* methylation analysis.

## Teaser

This diagnostic study examines the feasibility of screening newborns for the chromosome 15 imprinting disorders Angelman syndrome, Prader-Willi syndrome, and chromosome 15 duplication syndrome using *SNRPN* methylation analysis.

## Introduction

Chromosome 15 imprinting disorders, comprising Angelman syndrome (AS), Prader-Willi syndrome (PWS), and chromosome 15 duplication syndrome (Dup15q), are caused by deletions, duplications, or epimutations at the same imprinted region located at chromosome 15q11-q13.^1,2^ The 3 conditions have distinct phenotypes, but intellectual disability, aberrant behaviors, and social communication deficits are shared features.^3,4^ The promoter of the *SNRPN* gene, at the 15q11.2 locus, is differentially methylated according to the parent of origin, with the maternal copy being methylated and the paternal copy unmethylated. This feature is used routinely in the molecular diagnosis of AS and PWS. *SNRPN* promoter is usually unmethylated in AS owing to deletion of the maternal copy, paternal uniparental disomy of chromosome 15, or an imprinting defect of the maternal locus.^2^ In contrast, in PWS, *SNRPN* promoter is usually 100% methylated owing to paternal deletion, maternal uniparental disomy of chromosome 15, or an imprinting defect of the paternal allele.^5,6^ Dup15q results from duplications or triplications of the PWS or AS imprinted region, with triplication resulting from a supernumerary chromosome (isodicentric 15 [idic15]) and duplication resulting from an interstitial tandem duplication,^2^ resulting in differential *SNRPN* promoter methylation according to the parent of origin and the number of additional copies.

The advent of new genetic technologies and potential therapies has led to renewed interest in newborn screening for rare disorders,^7^ including genetically determined neurodevelopmental disorders for which early detection may benefit affected newborns and their families. Individuals affected by PWS, AS, or Dup15q may benefit from newborn screening followed by early targeted interventions that may become available in the next 5 years.^8,9,10^ For PWS, diagnosis in infancy allows for early initiation of growth hormone treatment to improve long-term outcomes.^11^ For AS and Dup15q, most infants do not receive a diagnosis that would allow intervention in the first year of life. Such early diagnosis, if available through newborn screening, could prevent the diagnostic odyssey, reducing medical costs and the significant stress and anxiety currently experienced by families while they await a diagnosis. For AS, there is now also an impending treatment aiming to reactivate *UBE3A* in clinical trials.^12^ A similar treatment has been recently developed for spinal muscular atrophy, resulting in this condition now being added to state-sponsored newborn screening programs.^13^

This unmet need has been recognized by 2 previous studies that proposed utility of *SNRPN* promoter methylation as a first-tier test for PWS and/or AS newborn screening.^14,15^ These studies used a small number of samples from PWS and/or AS patients and controls, primarily focused on methods of DNA extraction from blood spots, but did not examine method feasibility at population scale or utility for detection of infants with Dup15q. The primary objective of this study was to examine the feasibility of employing and implementing a novel workflow utilizing a first-tier high-throughput, low-cost screening test called methylation-specific quantitative melt analysis (MS-QMA) to quantify abnormal levels of *SNRPN* promoter methylation at population scale required for newborn screening for these conditions. It was hypothesized that it is feasible to perform newborn screening for chromosome 15 imprinting disorders using quantitative analysis of *SNRPN* promoter methylation as a first-tier test at a population scale.

## Methods

### Participants and Ethics

In this diagnostic screening study, participants for the test validation cohort were recruited as part of the FREE FX study^3,16^ and through pathology and clinical genetics services between January 2000 and December 2016 as detailed in eAppendix 1 in the Supplement. Data collection included retrospectively retrieved newborn blood spots (NBS) and dried blood spots (DBS) made at time of recruitment, as well as venous blood, buccal epithelial cells, and saliva samples for individuals with PWS (109 samples), AS (48 samples), or Dup15q (9 samples) and controls (1190 samples) from the general population. Data on race and ethnicity were not available for NBS samples from controls consented for deidentified research and most individuals affected with PWS, AS, and dup15q included in this study. These samples were used to validate MS-QMA as a first-tier test, establishing optimal *SNRPN* promoter methylation thresholds to make positive calls. Participants’ parents or guardians, and those participants who were cognitively able, provided written informed consent or had a legally acceptable representative provide consent. The test cohort included NBS consented for deidentified research from a population sample of 16 579 infants. These samples were used to examine feasibility of screening using MS-QMA at population scale. All study procedures were approved by the Royal Children’s Hospital Human Research Ethics Committee on May 24, 2013. This study followed the Strengthening the Reporting of Observational Studies in Epidemiology (STROBE) reporting guideline.

### Overview of the Testing Workflow

**Figure 1.**
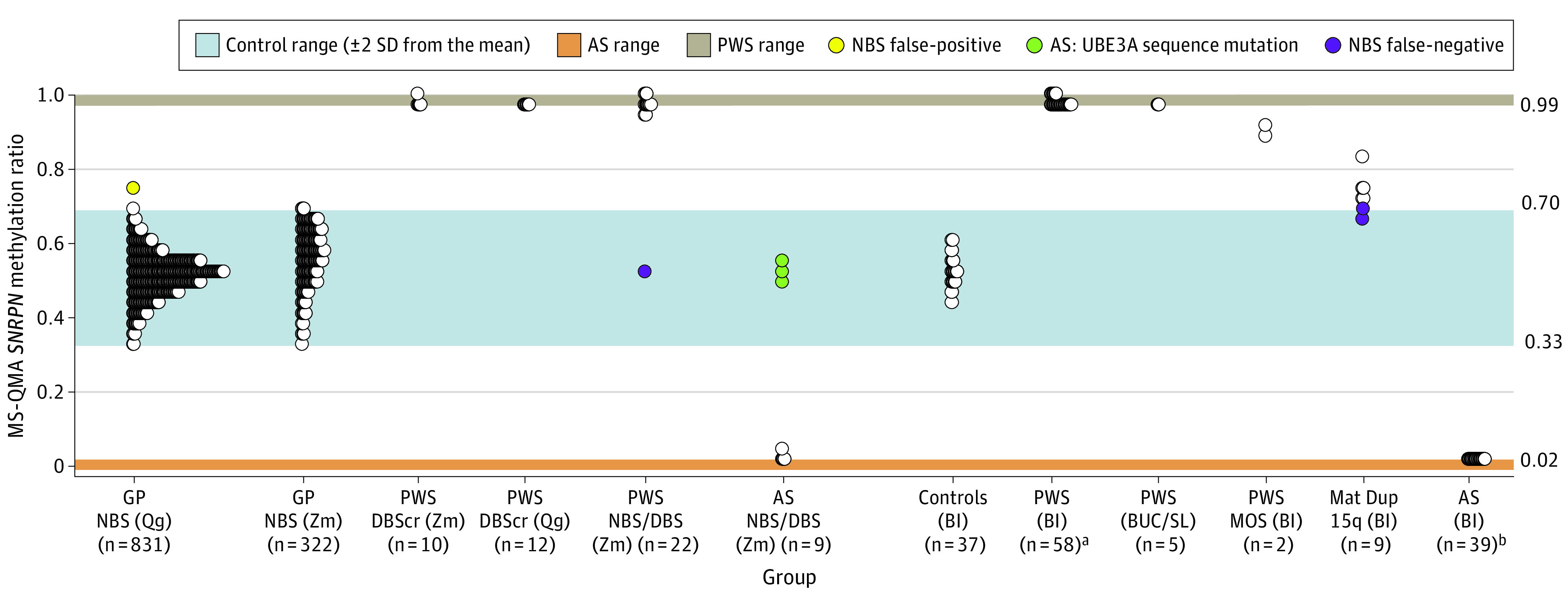
Validation of *SNRPN* Promoter Methylation Analysis Using Methylation Specific Quantitative Melt Analysis (MS-QMA) on 1356 Samples To monitor variability between runs, each 96-well plate had the following controls: (1) a dried blood spot sample from the same Prader-Willi syndrome (PWS) control (denoted as PWS DBScr) and Angelman syndrome (AS) and PWS–spiked DNA samples. NBS indicates newborn blood spots from the general population, showing comparison between Qiagen’s (Qg) and Zymo’s (Zm) bisulfite conversion systems. Of the 22 PWS NBS/DBS samples, 10 were from NBS, and 12 were from DBS made at time of recruitment. Of the 10 AS NBS/DBS samples, 3 were from NBS, and 7 were from DBS made at time of recruitment. Blood (BL), buccal epithelial cell (BUC), and saliva (SL) DNA had high quality from standard diagnostic testing for chromosome 15 imprinting disorders. Mat indicates maternal; MOS, mosaic PWS confirmed through standard diagnostic testing. ^a^Samples from a total of 44 individuals with AS, with 2 not overlapping between the AS (BL) and AS (NBS/DBS) groups. ^b^Samples from a total of 72 individuals with PWS, with 7 not overlapping between the PWS (BL) and PWS (NBS/DBS) groups.

The testing workflow consisted of first-tier high-throughput, low-cost screening using MS-QMA that detected abnormal levels of *SNRPN* promoter methylation applied to screen for chromosome 15 imprinting disorders in 16 579 infants consented for deidentified research as detailed in eFigure 1 and eAppendix 1 in the Supplement. All DNA and lysate from DBS samples (60-150 ng of DNA per sample analyzed using MS-QMA) were treated with sodium bisulfite prior to downstream methylation analysis. Bisulfite conversions were performed using either manual conversion with the EZ-96 DNA Methylation-Gold kit (Zymo Research), or automated conversion utilizing the EpiTect Bisulfite Kit (Figure 1) and QIAcube HT benchtop automation system (Qiagen), with MS-QMA first-tier testing performed as described in eAppendix 2 in the Supplement. The second-tier testing was performed on NBS-positive samples by MS-QMA and involved *SNRPN* promoter methylation analysis using competitive priming initiated nested quantification (CINQ) by droplet digital polymerase chain reaction (ddPCR) and copy number variation (CNV) analysis using the real-time PCR. CINQ ddPCR was performed using a total of 1-10 ng of purified DNA from diagnostic samples and controls or 20 μl of DBS lysate per sample, which were initially bisulfite converted, then amplified using CINQ ddPCR chemistry (eFigure 2 in the Supplement) and analyzed as described in eAppendix 3 in the Supplement. Copy number variation analysis was performed with the ViiA 7 Real-Time PCR System (Thermo Fisher Scientific) by using the real-time PCR relative standard curve method, normalized to β-globin copy number (2-copy control) as described in eAppendix 4 in the Supplement. Low-coverage whole-genome sequencing (LC-WGS)^30^ third-tier testing was performed only on samples that were positive by both first- and second-tier testing *SNRPN *CNV and methylation analyses as detailed in eAppendix 5 in the Supplement.

### Statistical Analyses

For the test cohort, positive predictive values (PPVs) were defined as the probability that individuals with a positive NBS MS-QMA test result truly have a molecular diagnosis of a disorder as confirmed using second- and third-tier testing. Specifically, this was the number of NBS samples that were positive by MS-QMA confirmed to be positive by second- and third-tier testing divided by the total number of NBS samples that were positive by MS-QMA (using specific thresholds detailed in eTable 1 in the Supplement). The prevalence was defined as the number of confirmed positive NBS samples divided by total number of NBS samples analyzed using MS-QMA. The confidence intervals for prevalence estimates were calculated using binomial distribution,^17^ conducted using Stata software, version 16 (StataCorp LLC). Data analyses were conducted between February 12, 2015, and August 15, 2020.

## Results

### Development and Validation of First-Tier Screening Protocol

Methylation-specific quantitative melt analysis of the *SNRPN* promoter methylation was developed using DNA samples from individuals with PWS and AS, spiked at different ratios (eFigure 3 in the Supplement) and then applied to 1356 samples, including NBS from the general population (eAppendix 6 in the Supplement). This cohort included NBS, DBS, and venous blood, buccal epithelial cells, and saliva samples from 77 individuals with PWS (median age, 3.00 years [IQR, 0.01-44.5 years]; 38 male [51.4%]), 46 individuals with AS (median age, 2.76 years [IQR, 0.028-49.00 years]; 21 male [45.5%]), and 9 individuals with maternal Dup15q (median age, 4.00 years [IQR, 1.00-28.00 years]; 6 male [66.7%]) (eTables 2-4 in the Supplement), corresponding to the results presented in Figure 1. Nine patients (6 PWS and 3 AS) whose samples were also analysed using MS-QMA did not have age reported. These patients were not included in the calculations of the percentage of males in each group. For this validation data set, MS-QMA showed a sensitivity of 99.0% for PWS, 93.8% for AS, and 77.8% for Dup15q; specificity of 100% for PWS, AS, and Dup15q; a PPV and negative predictive value (NPV) of 100% each for PWS and AS; and a PPV of 87.5% and NPV of 100% for Dup15q (eFigure 4 in the Supplement).

### First-Tier Screening on NBS From 16 579 Infants

**Figure 2.**
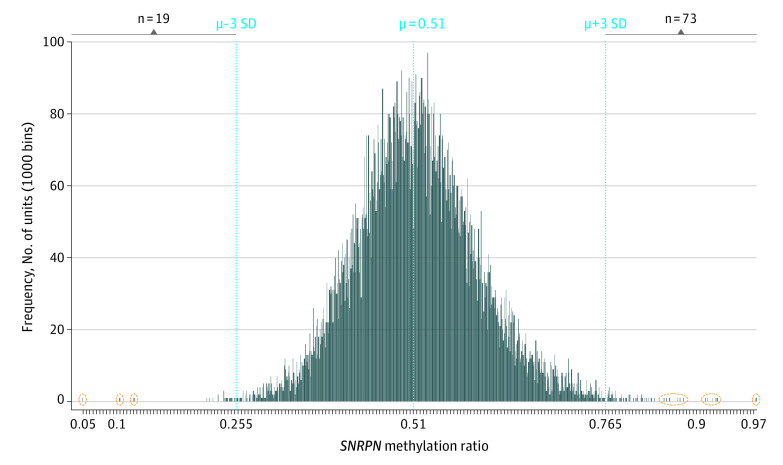
Quantitative Analysis of *SNRPN* Promoter Methylation Using MS-QMA First-Tier Testing on Newborn Blood Spots (NBS) From 16 579 Infants Frequency distribution histogram from 16 579 NBS samples, with each vertical bar representing NBS methylation values. While the mean (μ), minimum, and maximum values (2 SDs from the mean) in this larger data set were almost identical to those in the initial validation data set (Figure 1), the spread of tails at both ends of the distribution for this larger data set was greater. Subsequently, the minimum and maximum cut-off values of the normal distribution for the larger data set were increased to 3 SDs from the mean for calling of positive cases. Vertical broken lines represent the mean methylation value and +/− 3 SDs from the mean; the n values indicate the number of positive cases above or below these upper and lower normal distribution values; and extreme outliers at either end of the distribution reflexed for second-tier testing are circled in orange.

**Figure 3.**
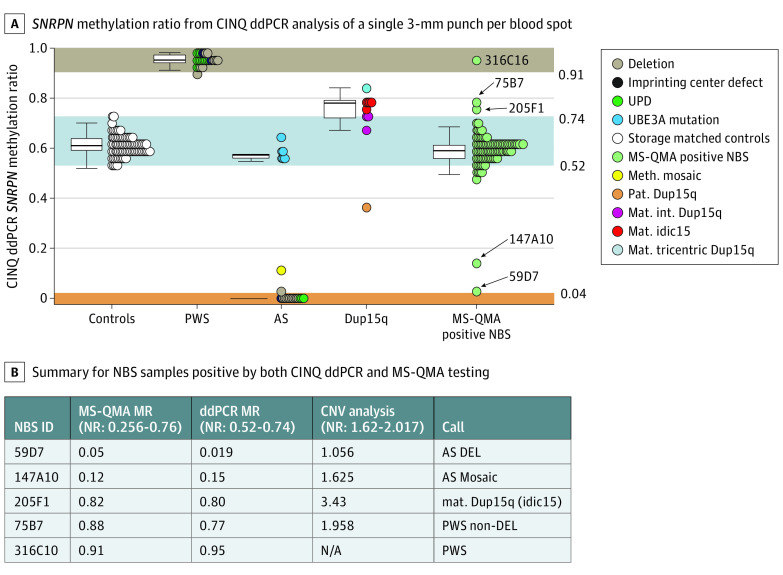
Confirmatory *SNRPN* Promoter Methylation Testing Using Competitive Priming Initiated Nested Quantification Using Droplet Digital PCR (CINQ ddPCR) on Newborn Blood Spot (NBS) Samples Tested Positive by First-Tier MS-QMA Screening A, *SNRPN* methylation ratio from CINQ ddPCR analysis of a single 3 mm punch per blood spot, including 20 storage-matched control NBS samples (from the general population with MS-QMA methylation ratio of 0.5), dried blood spot samples from 25 patients with PWS, 22 patients with AS, and 11 patients with Dup15q syndrome identified as part of standard diagnostic testing, as well as 92 NBS samples that tested positive by MS-QMA analysis, as part of first-tier testing. NBS IDs are included for the 5 samples confirmed to have abnormal *SNRPN* promoter methylation. B, Summary of NBS samples that were positive by both CINQ ddPCR and MS-QMA testing from Figure 2 and results on the *SNRPN* copy number variation (CNV) analysis from Figure 4, with calls made based on neurotypical control reference ranges (NR) and methylation ratio reference ranges in panel A from DBS samples from patients with diagnosis confirmed by standard of care diagnostic testing. N/A indicates value not available.

**Figure 4.**
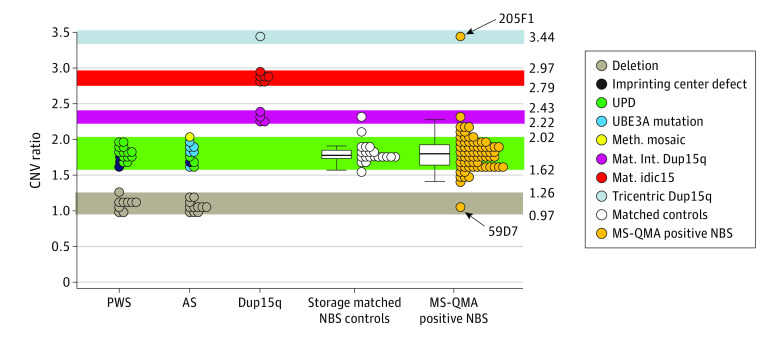
Confirmatory *SNRPN* Copy Number Variation (CNV) Testing Using Real-Time Polymerase Chain Reaction (PCR) Relative Standard Curve Method on Newborn Blood Spot (NBS) Samples That Were Positive by First-Tier MS-QMA Testing Of the 92 NBS samples shortlisted from first-tier testing that were tested by CINQ ddPCR, sufficient DNA was available for only 69 NBS samples for CNV confirmatory testing. *SNRPN* CNV analysis was performed using real-time PCR relative standard curve method of a single 3 mm punch per blood spot, including dried blood spot (DBS) samples from 25 patients with PWS, 22 patients with AS, and 11 patients with Dup15q syndrome identified as part of standard diagnostic testing, as well as 20 storage-matched control NBS samples (from the general population with MS-QMA methylation ratio of 0.5) and 69 NBS samples that were positive by quantitative MS-QMA analysis. For all DBS samples used to establish CNV reference ranges, the copy number of the PWS/AS imprinted region was confirmed in diagnostic settings using chromosomal microarray. The 1-copy reference range (highlighted in brown) between 0.97 and 1.26 CNV ratio units was established using minimum and maximum values from 20 deletion cases (PWS and AS deletion groups collapsed). The 2-copy reference range (highlighted in green) between 1.62 and 2.02 CNV ratio was established using minimum and maximum values from 27 nondeletion cases (PWS and AS nondeletion groups collapsed). The 4- and 5-copy reference ranges (highlighted in purple and gray) were established using minimum and maximum values from DBS samples from 4 interstitial 15q duplication and 6 isodicentric 15q patients. Although the 7-copy reference range could not be established because there was only 1 patient with tricentric 15q, the reference value for that 1 DBS sample has been included (highlighted in light blue). Black arrows point to 2 samples with the highest and lowest values overlapping with tricentric 15q and deletion CNV ranges that were reflexed to LC-WGS confirmatory testing.

Methylation-specific quantitative melt analysis was then applied to 16 579 NBS samples consented for deidentified research (Figure 2). In contrast to blood spots used for MS-QMA validation that were stored for less than 1 year (Figure 1), NBS samples from the larger cohort had been stored for longer than 5 years at room temperature prior to MS-QMA analysis, except for 830 NBS samples that were included in both validation and test cohorts. *SNRPN* promoter methylation analysis using MS-QMA in 16 579 NBS identified 92 cases (0.55%) (eTable 1 in the Supplement) with values outside the normal range (3 standard deviations from the mean), which were referred for confirmatory testing (Figures 3 and 4). There was no significant difference in *SNRPN *promoter methylation between sexes in this cohort (eTable 5 in the Supplement) with the numbers and statistics for samples analyzed summarized in eTable 6 in the Supplement.

### Development and Application of Second-Tier Methylation-Based Confirmatory Testing

CINQ ddPCR for second-tier testing was developed and validated on high-quality DNA isolated for diagnostic testing (eFigure 5 in the Supplement) and showed 100% sensitivity and specificity to differentiate blood spot and venous blood DNA of 33 individuals affected with chromosome 15 imprinting disorders from 44 neurotypical controls (eAppendix 7 in the Supplement). Of the 92 NBS samples that were positive by MS-QMA analysis, only 5 samples had a methylation ratio outside the normal reference range and were called as confirmed positive cases by CINQ ddPCR. Raw CINQ ddPCR data from 2-D plots from these positive samples (eFigure 6 in the Supplement) were used to support these calls.

### Second-Tier CNV Locus Specific Confirmatory Testing

**Figure 5.**
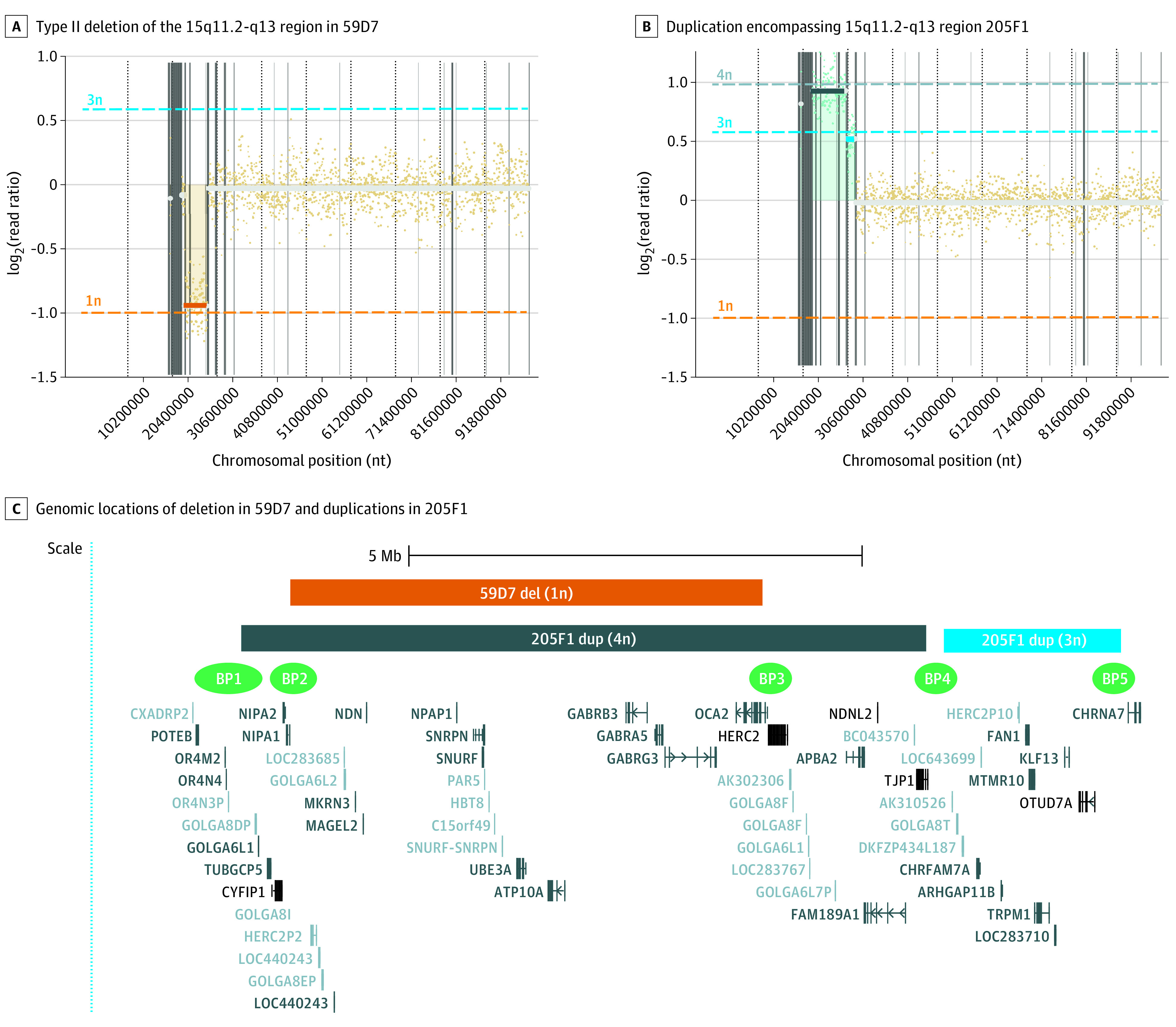
Confirmatory Testing Using Low-Coverage Whole Genome Sequencing (LC-WGS) LC-WGS analysis on newborn blood spot (NBS) samples that tested positive by first- and second-line testing, with the lowest and highest copy number variation results of the shortlisted samples, confirming that A, 59D7 is a typical Angelman syndrome case caused by the type II deletion of the 15q11.2-q13 region between BP2 and 3 highlighted by orange rectangle; and that B, 205F1 is an idic15 case, with larger duplicated region encompassing 15q11.2-q13 PWS/AS imprinted center highlighted by the dark blue rectangle; the proximal region is highlighted by light blue rectangle, encompassing an additional gene cluster proximal to *CHRNA7*, with specific locations and gene names included in panel C. Approximate locations of common breakpoints BP1 to BP5 are indicated in green.^18,19^ In panels A and B, the gray, light blue, and orange lines represent the thresholds for the relative numbers of 4 copies, 3 copies, and 1 copy, respectively. The gray line with the log_2_(ratio) of 0 (y-axis) represents the threshold for the relative number of 2 copies. BP indicates breakpoint.

Copy number variation analysis was validated on DBS samples from 25 patients with PWS, 22 patients with AS, and 11 patients with Dup15q identified as part of standard diagnostic testing and on 20 control NBS samples (from the general population with MS-QMA methylation ratio of 0.5). These controls were storage-matched for the samples shortlisted as part of first-tier screening by MS-QMA. The analysis was co-run with 69 NBS samples shortlisted from the first-tier MS-QMA screen that had sufficient lysate available following CINQ ddPCR analysis (Figure 4). For positive and negative controls, the CNV analysis showed sensitivity and specificity approaching 100% to differentiate (1) PWS, AS, and isodicentric and tricentric Dup15q from one another and from the NBS controls and (2) deletion from nondeletion PWS and AS groups and from isodicentric and tricentric Dup15q subtypes. The CNV ratio for only 1 archival control NBS overlapped with the interstitial Dup15q range, suggesting that long-term storage on archival NBS samples contributed to variability in the CNV ratio. For the 69 NBS samples shortlisted from the first-tier MS-QMA screen, the assay identified 2 distinct outliers, one within the deletion range (59D7) and the other in the tricentric Dup15q range (205F1). These 2 samples were reflexed for LC-WGS third-tier testing to confirm these CNV results (Figure 5).

### Development of Third-Tier Confirmatory Testing Using LC-WGS

For patient samples that returned a positive CNV test result, routine analysis would be to collect an additional DNA sample to perform a copy number analysis using a chromosomal microarray or a similar platform. However, owing to the restricted material available for each sample and DNA quality issues related to long-term storage at room temperature in this study, array-based approaches were not feasible. As an alternative, we used low-input LC-WGS to identify chromosomal CNVs from a single 3 mm NBS punch as input. Low-coverage whole-genome sequencing was developed and validated on DBS samples from individuals with a confirmed diagnosis through standard diagnostic testing and controls from the general population. This method effectively differentiated between idic15, deletion, and control samples with 100% specificity and sensitivity, using lysate from a single 3 mm punch per blood spot. Moreover, this method could be used to differentiate breakpoints for DBS samples from idic15 cases (eFigure 7 in the Supplement).

### Application of Third-Tier Confirmatory Testing Using LC-WGS

The 2 samples that returned the highest and lowest CNV ratio results (Figure 4) were investigated using LC-WGS. Case 59D7 showed typical AS caused by the deletion of the 15q11.2-q13 region (NC_000015.9:g.23100001_28300000del). Case 205F1 showed a supernumerary duplication encompassing 2 copies of the 15q11.2-q13 with the duplication extending to 15q13.3 (NC_000015.9:g.22550001_30100000[4]; NC_000015.9:g.30300001_32250000[3]). This duplication encompassed a gene cluster proximal to *CHRNA7* but at a lower coverage, suggesting the presence of 3 copies for this region relative to the estimated 4 copies for the 15q11.2-q13 imprinted region (Figure 5). This observation is consistent with 205F1 being idic15, with 1 breakpoint at BP4 and 1 breakpoint at BP5. This is a common arrangement previously reported for idic15^18,19^ and is consistent with the LC-WGS validation data set for the idic2 panel in eFigure 7B in the Supplement.

### Positive Predictive Values for MS-QMA First-Tier Screening

We determined PPVs and prevalence estimates from quantitative methylation analysis performed on 16 579 NBS samples (eTable 1 and eFigure 8 in the Supplement) using 2 sets of thresholds. Use of less conservative thresholds (0.255 to 0.765 methylation ratio; mean [±3 SD] methylation ratio, 0.51 [0.255]) for the MS-QMA first-tier testing resulted in PPVs of 10.0%, 2.7%, and 1.1% for AS, PWS, and Dup15q, respectively. Use of more conservative thresholds from Figure 1 on DBS samples with PWS and AS diagnosis confirmed in diagnostic settings (PWS methylation ratio ≥0.88; AS methylation ratio ≤0.12) resulted in PPVs of 67.0% for AS, 33.0% for PWS, and 44.0% for all chromosome 15 imprinting disorders combined for the validation cohort. Although the use of more conservative thresholds improved these PPVs, it prevented MS-QMA from detecting 1 Dup15q case.

## Discussion

We have demonstrated the feasibility of a workflow using quantitative analysis of *SNRPN* promoter methylation as a first-tier test to screen for chromosome 15 imprinting disorders using NBS from an unbiased newborn cohort at a population scale. The preliminary prevalence estimates for AS and PWS were at 1 in 8290, and for Dup15q syndrome at 1 in 16 579. These figures for AS and PWS are consistent with a Danish study reporting a prevalence of 1 in 10 000 for AS^20^ but are higher than those previously reported for PWS in biased cohorts with prevalence ranging between 1 in 15 000^21^ and 1 in 30 000.^22,23^ The MS-QMA first-tier testing showed relatively high PPVs for molecular diagnosis of AS and PWS in line with the first-tier testing currently included in state-sponsored newborn screening programs. The MS-QMA PPVs were determined in this study test cohort to be 67% for AS, 33% for PWS, and 44% for all chromosome 15 imprinting disorders combined. For comparison, PPVs for many of the conditions included in the state-sponsored newborn screening programs range between 0.5% and 67%.^24,25,26^ Having high PPVs is important for newborn screening, because it ensures that (1) there is a lower number of false-positive results that need to be repeated, leading to lower overall laboratory costs; (2) there is less work for maternity services in obtaining a repeat blood sample for the majority of cases flagged as potential positives; and (3) there is minimized psychological effect from false-positive calls for the families contacted as part of the follow-up.

### Importance of Quantitative Methylation Analysis as Part of First-Tier Screening

Detection of all 5 probands with confirmed molecular diagnoses was only made possible in this study by the inclusion of quantitative methylation analysis as a first-tier test, wherein distinct positive and negative thresholds could be applied in an objective manner. Another study using a high-resolution melt–based method suggested exclusive use of qualitative subjective analysis of derivative curves for newborn screening of chromosome 15 imprinting disorders (trialed on only a small number of samples).^15^ From the large population-scale high-resolution melt data set used for MS-QMA analysis, we found that this subjective approach would not have identified 4 of the 5 probands confirmed to be positive in this study (eFigure 8 in the Supplement).

It is important to also note that quantitative analysis using MS-QMA was initially developed for newborn screening of fragile X syndrome in both sexes, targeting *FMR1* promoter methylation,^27,28^ but was modified to also detect *SNRPN* promoter methylation in this study. This modification sets the proposed approach apart from other methods^14,15^ because MS-QMA has the potential to simultaneously screen newborns for chromosome 15 imprinting disorders and fragile X syndrome, as well as other rare disorders with distinct methylation signatures.^29^

### Reagent Cost and Sample Requirements for First-Tier Newborn Screening

One important reason why chromosome 15 imprinting disorders are not included in current newborn screening programs is the lack of a first-tier test that has low laboratory costs. The test also must work on one or two 3 mm punches of NBS material that may be of poor quality, which may be problematic for genetic or genomic testing technologies that require DNA extraction with DNA of much higher concentration and quality (that in itself would make the test too expensive at approximately US$10 per extraction), such as chromosomal microarray, multiplex ligation-dependent probe amplification,^14^ and methylation-specific high-resolution melt.^15^

For comparison, the reagent cost to simultaneously test for the 3 conditions (PWS, AS, and Dup15q) on a single 3 mm punch was less than US$4 in this study, or less than US$1.3 per condition per infant. This cost was made possible through use of high-throughput crude DNA extraction (no commercial kits used) in 96-well format, automated bisulfite conversion using the QIAcube HT system (Qiagen), and low volumes for MS-QMA screening (5 μl reactions, 96 at a time) to further minimize the reagent cost.

### Limitations

The primary limitation of this study is that the identified probands could not be followed up to assess their phenotype. Lack of follow-up meant that the positive results could not be confirmed on another tissue sample in diagnostic settings. The deidentified nature of the study also precluded any estimates of the false negative rate of screening. However, comparison of the prevalence estimates from our study with reported estimates suggest that this rate would be low. Another limitation is that AS, owing to a *UBE3A* sequence mutation, shows normal *SNRPN* promoter methylation approaching 50% and could not be differentiated by MS-QMA from general population controls. This suggests that the AS estimates provided might not be totally accurate, given that 10% of AS cases are thought to be caused by *UBE3A* mutations.^2^ Moreover, although the use of more conservative thresholds significantly improved PPVs for AS and PWS, it prevented detection of the maternal Dup15q case, which may be considered as another limitation. However, with fresher samples in future prospective studies, limitations associated with DNA quality issues and use of less conservative thresholds on results from archival blood spots would be addressed, as in Figure 1. Moreover, cases positive by MS-QMA will be reflexed for second- and third- tier testing on another NBS punch, with the clinical proof of Dup15q coming from LC-WGS to further increase diagnostic yield for Dup15q.

## Conclusions

In summary, this diagnostic study demonstrated that screening for all chromosome 15 imprinting disorders at a population scale was feasible using quantitative analysis of *SNRPN* promoter methylation, with the reagent cost, sample requirements, prevalence, and PPV estimates compatible with screening for other conditions in the state sponsored programs. If these findings and the preliminary prevalence estimates are confirmed in larger future prospective studies, this workflow could ensure that early interventions for these disorders are uniformly available to most infants from birth as part of state-sponsored newborn screening programs.

## Notes

Supplement. **eAppendix 1.** Participant recruitment **eAppendix 2.** MS-QMA testing **eAppendix 3.** CINQ ddPCR testing **eAppendix 4.** CNV analysis **eAppendix 5.** Low Coverage – Whole Genome Sequencing (LC-WGS): 3rd-tier testing **eAppendix 6.** Validation of 1st-tier DNA methylation-based screening **eAppendix 7. **Validation of Competitive Priming Initiated Nested Quantification (CINQ) using droplet digital PCR (ddPCR) **eTable 1.** Positive predictive values (PPV) and incidence estimates for different MS-QMA thresholds from quantitative methylation analysis of 16,579 NBS **eTable 2. **Demographic information and *SNRPN* methylation ratio (MR) comparisons of the samples from the validation cohort **eTable 3. **Demographic information and *SNRPN* methylation ratio (MR) for confirmatory testing of PWS and AS samples stratified by tissue type from the validation cohort **eTable 4. **Distribution of *SNRPN* Methylation Ratio (MR) and comparisons between sexes for the NBS samples from the general population from the MS-QMA validation cohort **eTable 5. **Distribution of *SNRPN* Methylation Ratio (MR) and comparisons between sexes for the NBS samples from the general population from the MS-QMA test cohort **eTable 6.** Summary of the numbers and statistics for the samples analysed for *SNRPN *methylation levels using MS-QMA in newborn blood spots (NBS) from 16,579 infants **eFigure 1.** Screening and confirmatory testing workflow developed for prevalence studies on newborn blood spots consented for de-identified research **eFigure 2.** Competitive Priming Initiated Nested Quantification using droplet digital PCR (CINQ ddPCR) **eFigure 3.** Assessing variability in *SNRPN* methylation output between 8 runs using the same control spiked lymphoblast DNA samples on each plate **eFigure 4.** CINQ droplet digital PCR validation for quantitative analysis of *SNRPN* methylation **eFigure 5.** Examples of 2-D plots from CINQ ddPCR for NBS confirmed to have abnormal *SNRPN* methylation co-run with DBS from positive and negative controls **eFigure 6.** Validation of confirmatory testing using Low-Coverage Whole Genome Sequencing (LC-WGS) **eFigure 7.** Summary of newborn blood spots tested, and the positive cases identified by 1^st^, 2^nd^, and 3^rd^-tier testing **eFigure 8.** Derivative curve profiles of 5 cases positive by quantitative methylation analysis using MS-QMA
