# Supplementary material for: Feasibility of Screening for Chromosome 15 Imprinting Disorders in 16 579 Newborns by Using a Novel Genomic Workflow
Source: JAMA Netw Open. 2022 Jan 4;5(1):e2141911. doi: 10.1001/jamanetworkopen.2021.41911 (PMC8728620; doi:10.1001/jamanetworkopen.2021.41911)
Supplement: Supplement. — eAppendix 1. Participant recruitment eAppendix 2. MS-QMA testing eAppendix 3. CINQ ddPCR testing eAppendix 4. CNV analysis eAppendix 5. Low Coverage – Whole Genome Sequencing (LC-WGS): 3rd-tier testing eAppendix 6. Validation of 1st-tier DNA methylation-based screening eAppendix 7. Validation of Competitive Priming Initiated Nested Quantification (CINQ) using droplet digital PCR (ddPCR) eTable 1. Positive predictive values (PPV) and incidence estimates for different MS-QMA thresholds from quantitative methylation analysis of 16,579 NBS eTable 2. Demographic information and SNRPN methylation ratio (MR) comparisons of the samples from the validation cohort eTable 3. Demographic information and SNRPN methylation ratio (MR) for confirmatory testing of PWS and AS samples stratified by tissue type from the validation cohort eTable 4. Distribution of SNRPN Methylation Ratio (MR) and comparisons between sexes for the NBS samples from the general population from the MS-QMA validation cohort eTable 5. Distribution of SNRPN Methylation Ratio (MR) and comparisons between sexes for the NBS samples from the general population from the MS-QMA test cohort eTable 6. Summary of the numbers and statistics for the samples analysed for SNRPN methylation levels using MS-QMA in newborn blood spots (NBS) from 16,579 infants eFigure 1. Screening and confirmatory testing workflow developed for prevalence studies on newborn blood spots consented for de-identified research eFigure 2. Competitive Priming Initiated Nested Quantification using droplet digital PCR (CINQ ddPCR) eFigure 3. Assessing variability in SNRPN methylation output between 8 runs using the same control spiked lymphoblast DNA samples on each plate eFigure 4. CINQ droplet digital PCR validation for quantitative analysis of SNRPN methylation eFigure 5. Examples of 2-D plots from CINQ ddPCR for NBS confirmed to have abnormal SNRPN methylation co-run with DBS from positive and negative controls eFigure 6. Validation of confirmator [file jamanetwopen-e2141911-s001.pdf]

Supplemental Online Content

Godler DE, Ling L, Gamage D, et al. Feasibility of screening for chromosome 15 imprinting disorders in 16 579 newborns by using a novel genomic workflow. *JAMA Netw Open.* 2022;5(1):e2141911. doi:10.1001/jamanetworkopen.2021.41911

eAppendix 1. Participant recruitment

eAppendix 2. MS-QMA testing

eAppendix 3. CINQ ddPCR testing

eAppendix 4. CNV analysis

eAppendix 5. Low Coverage – Whole Genome Sequencing (LC-WGS): 3rd-tier testing

eAppendix 6. Validation of 1st-tier DNA methylation-based screening

eAppendix 7. Validation of Competitive Priming Initiated Nested Quantification (CINQ) using droplet digital PCR (ddPCR)

eTable 1. Positive predictive values (PPV) and incidence estimates for different MS-QMA thresholds from quantitative methylation analysis of 16,579 NBS

eTable 2. Demographic information and *SNRPN* methylation ratio (MR) comparisons of the samples from the validation cohort

eTable 3. Demographic information and *SNRPN* methylation ratio (MR) for confirmatory testing of PWS and AS samples stratified by tissue type from the validation cohort

eTable 4. Distribution of *SNRPN* Methylation Ratio (MR) and comparisons between sexes for the NBS samples from the general population from the MS-QMA validation cohort

eTable 5. Distribution of *SNRPN* Methylation Ratio (MR) and comparisons between sexes for the NBS samples from the general population from the MS-QMA test cohort

eTable 6. Summary of the numbers and statistics for the samples analysed for *SNRPN* methylation levels using MS-QMA in newborn blood spots (NBS) from 16,579 infants

eFigure 1. Screening and confirmatory testing workflow developed for prevalence studies on newborn blood spots consented for de-identified research

eFigure 2. Competitive Priming Initiated Nested Quantification using droplet digital PCR (CINQ ddPCR)

© 2022 Godler DE et al. *JAMA Network Open.*

eFigure 3. Assessing variability in *SNRPN* methylation output between 8 runs using the same control spiked lymphoblast DNA samples on each plate

eFigure 4. CINQ droplet digital PCR validation for quantitative analysis of *SNRPN* methylation

eFigure 5. Examples of 2-D plots from CINQ ddPCR for NBS confirmed to have abnormal *SNRPN* methylation co-run with DBS from positive and negative controls

eFigure 6. Validation of confirmatory testing using Low-Coverage Whole Genome Sequencing (LC-WGS)

eFigure 7. Summary of newborn blood spots tested, and the positive cases identified by 1st, 2nd and 3rd -tier testing

eFigure 8. Derivative curve profiles of 5 cases positive by quantitative methylation analysis using MS-QMA

This supplemental material has been provided by the authors to give readers additional information about their work.

© 2022 Godler DE et al. *JAMA Network Open*.

eAppendix 1. Participant recruitment and workflow overview.

The validation cohort (eFigure 1) for first tier *SNRPN* methylation screening using methylation specific quantitative melt analysis (MS-QMA) included 1,153 NBS consented for de-identified research at Victorian Clinical Genetics Services (VCGS) in 2016, 12 DBS and NBS from individuals with confirmed diagnosis of PWS, and 9 with diagnosis of AS recruited into the FREE FX study, as well as, de-identified good quality DNA from blood, buccal epithelial cells and saliva, of 37 neuro-typical controls, 65 PWS, 39 AS and 9 maternal Dup15q patients with diagnoses confirmed using chromosomal microarray and *SNRPN* methylation diagnostic testing at VCGS, INTA University of Chile, Greenwood Genetic Center and University of Padua. The second-tier methylation testing was performed using Competitive Priming Initiated Nested Quantification (CINQ) ddPCR. The development and validation cohort for CINQ ddPCR (Fig S2 and S3) included de-identified blood and saliva DNA samples from 44 neuro-typical controls, 38 PWS, 15 AS and 18 with Dup15q from the FREE FX study, VCGS and University of Kansas Medical Centre (USA). The CINQ ddPCR cohort of participants who provided blood for DBS control samples (examined within the same run with NBS reflexed for confirmatory testing), were from 25 PWS, 22 AS and 11 Dup15q syndrome patients recruited into the FREE FX study with molecular diagnosis confirmed in standard diagnostic settings. The Validation cohort for the CNV real-time PCR and LC-WGS analyses included FREE FX study participants: 25 PWS, 22 AS and 11 Dup15q, identified as part of standard diagnostic testing. They provided blood to generate DBS which were examined within the same run with NBS reflexed for confirmatory testing. The FREE FX study participants were recruited nationally through: (i) VCGS, Murdoch Children's Research Institute and Genetics of Learning Disability Service (GOLD), Hunter Genetics; (ii) referring practitioners; and (iii) family support groups. The infant test cohort utilized for prevalence studies included 15,749 NBS consented in 2011, and 830 consented in 2016 for de-identified research at part of

© 2022 Godler DE et al. *JAMA Network Open*.

the standard Newborn Screening Program at VCGS. While the study would have benefitted greatly from use of fresher samples in this test cohort, ethics approval did not permit for this to occur. The study was required to screen only the NBS consented for de-identified research from 2011 population sample because by 2016 (when the samples were screened in this study) most children from this cohort should have received their diagnosis by standard of care testing. This in turn minimized potential ethical issues associated with not returning the results for the probands identified from this study.

Briefly, for the test cohort all NBS were punched into 3 replicate 96 well plates (eFig 1). The first two plates had a single 3mm NBS punch per infant per well, while the third plate had three 3mm punches per well. The first plate was used for 1st-tier testing utilizing MS-QMA to analyze methylation of the *SRNPN* promoter. The second and third plates were used for precise quantitation of *SNRPN* promoter DNA methylation using CINQ ddPCR and a *SNRPN* real-time PCR CNV analysis to detect changes in copy number at the *SNRPN* locus. Samples confirmed by 2nd-tier testing to have an abnormal CNV result were referred to 3rd-tier testing involving LC-WGS from the third plate (eFig 1).

© 2022 Godler DE et al. *JAMA Network Open*.

eAppendix 2. MS-QMA testing.

Two separate bisulfite conversions were performed per patient sample, as previously described [18]. Ninety-six samples were bisulfite converted at a time (3 controls and 93 unknown samples per plate) and were serially diluted once post-conversion. Each set of four 96 well plates was then transferred into a 384 well format for real-time PCR analysis utilizing MeltDoctor™ high-resolution melt (HRM) reagents in 5 µl reactions, as per manufacturer's instructions (Life technologies, Foster City, CA). For real-time PCR, a unique primer set was used (E.D.G Innovations & Consulting, Melbourne Australia) that targets specific CpG sites within the *SNRPN* promoter. The annealing temperature for the thermal cycling protocol was 65 °C for 40 cycles. The ViiA™ 7 Real-Time PCR System (Life technologies, Foster City, CA) was then used to measure the rate of dye incorporation into double stranded DNA in order to quantify DNA concentration of the unknown samples following bisulfite conversion using the relative standard curve method. The dynamic linear range (between 0.05–10 ng/µl) was determined from the standard curve using a series of doubling dilutions of a converted DNA standard from a control lymphoblast cell line and embedded in the quality control (QC) parameters used by the Q-MAX software (Curve, Melbourne, Australia) for quantitative methylation analysis by MS-QMA.

The unknown samples that did not have DNA concentration post-bisulfite conversion within this dynamic linear range, were flagged by the Q-MAX software as not meeting this quality control parameter prior to the HRM analysis (that would follow in close tube format). The products from methylated and unmethylated *SNRPN* promoter sequences were then separated into single strands in the temperature range of 76.5°C and 84.5°C. The HRM software module for ViiA™ 7 System was then used to plot the rate of PCR product separation to single strands at different temperatures with the difference in fluorescence converted to aligned fluorescence units (AFU) at 80°C. The AFU conversion to the methylation percentage,

© 2022 Godler DE et al. *JAMA Network Open*.

and all of the above quality control steps, were analyzed simultaneously for 384 reactions at a time using Q-MAX software (Curve Tomorrow, Melbourne, Australia), developed to automate the process.

This software utilized a custom-designed computer algorithm to simultaneously perform multiple quality control checks to determine DNA concentrations and quality post-bisulfite conversion using raw real-time PCR data, as well as, uniformity of HRM profile data outputs between 4 technical replicates per sample (2 bisulfite reactions and 2 dilutions [1 per conversion]). The HRM data for the sample dilutions outside the QC ranges were automatically discarded and were not included inthe quantitative methylation analysis by the Q-MAX software, as previously described [18].

eAppendix 3. CINQ ddPCR testing.

Each ddPCR reaction consisted of 12.5µl of 2x Biorad Evaagreen Supermix (Bio-Rad Laboratories, Gladesville, Australia), 0.125µl of *SNRPN* Forward and Reverse primers external primers that amplified methylated and unmethylated sequences with equal efficiency; 0.375µl of forward and reverse internal methylation specific primers (E.D.G Innovations & Consulting; Vic, Australia) and 11.5µl of bisulfite converted DNA. The PCR-cycling conditions were 95 ºC for 5 minutes, 44 cycles of 94 ºC for 30 seconds, 60 ºC for 1 minute, 98 ºC for 5 minutes, followed by an indefinite hold at 12 ºC. Following generation of 20,000 droplets per sample, the samples were run on a Bio-Rad 200QX droplet digital systerms (Bio-Rad Laboratories, Gladesville, Australia), with data analyzed using the Bio-Rad QuantaSoft software (version 1.7.4.0917), where methylation ratio (MR) was calculated for each sample representing total number of positive droplets with methylation positive amplicons / total number of positive droplets. The relationship of observed MR with expected methylation percentage is shown in eFig 5B where 50% methylation equates to 0.7 MR.

© 2022 Godler DE et al. *JAMA Network Open.*

eAppendix 4. CNV analysis.

Probes for both assays were labelled with FAM, with *SNRPN* primers/probes designed by E.D.G Innovations & Consulting (VIC, Australia); and *β*-globin 2 copy control assay primers and probes from earlier studies [31]. The PCR reactions for both assays consisted of 5µl total volume, including 2X SensiFAST™ Probe Lo-ROX Mastermix (Bioline, London, UK), 0.2µl of 10 µmol/L either *SNRPN* or *β*-globin primers, 0.1µl of 10 µmol/L either *SNRPN* or *β*-globin Probe, 1 µl water and 1µl of DNA lysate. The RT PCR-cycling conditions were 95 ºC for 2 minutes, 44 cycles of 95 ºC for 10 seconds, 60 ºC for 20 seconds. The real-time PCR data were processed using the ViiA7 software (version 1.2). The *SNRPN* and β-globin dynamic linear range was performed on a series of doubling dilutions of a DNA standard (100 to 0.8 ng/µl). All samples were assayed in quadruplicate 10 µl single-plexed PCR reactions, with mean results from the technical replicates expressed in arbitrary units (au) in relation to the standard curves performed on each plate.

eAppendix 5. Low Coverage – Whole Genome Sequencing (LC-WGS): 3rd-tier testing.

LC-WGS was performed on DNA extracted from a single 3mm DBS punch per participant. The method utilized Nextera DNA Flex Library Prep as per manufacturer's instructions (Illumina, CA, USA), with sequencing performed on Illumina Novaseq (Illumina, CA, USA) at 2 X 150bp reads to a minimum of 50 million reads per sample. Reads were aligned to the human hg19 reference genome using BWA-mem and duplicates removed using Picard MarkDuplicates. Copy number analysis was performed using the WisecondorX[14] where test samples were compared a panel of 10 control samples extracted and sequenced in parallel. A bin size of 50kb was used for WisecondorX analysis and variants greater than 300kb with a ratio mean of less than 0.15 or greater than 0.15 were included in the analysis.

© 2022 Godler DE et al. *JAMA Network Open*.

eAppendix 6. Validation of 1st tier DNA methylation-based screening.

Inter-run variability was assessed for quantitative analysis of *SNRPN* promoter using MS-QMA on six DNA samples with different levels of *SNRPN* methylation achieved through spiking an AS sample showing 0% *SNRPN* methylation with a PWS sample showing 100% *SNRPN* methylation (eFig 3). The MS-QMA output was highly reproducible over the 8 runs at 0%, 87%, 95% and 100% methylation, with 2 standard deviations of 1%, 3%, 4% and 2%, respectively. As methylation levels approached 50%, the technical variability between runs increased, with 2 standard deviations of 8%.

The method was then applied to 1,356 samples. There was one false positive result (yellow dot Fig 1) and one PWS NBS and 2 Dup15 NBS showing a false negative results. All AS NBS/DBS cases other than those caused by a *UBE3A* sequence mutation, had methylation ratio approaching 0 and were correctly identified by MS-QMA to be in the AS methylation range. As expected, DBS from AS due to a *UBE3A* sequence mutation showed *SNRPN* methylation approaching 50% and could not be differentiated from general population controls.

Selection of bisulfite conversion method

The effect of two bisulfite conversion method on distribution of MS-QMA MR outputs was examined for 1,153 NBS from the general population. MS-QMA analysis values from the Qiagen method showed normal distribution, with mean approaching 50%, and minimum and maximum ‘normal range’ values at 0.33 and 0.7 MR respectively (defined based on being 2 standard deviations from the mean). While the MS-QMA analysis values from the Zymo Research method showed identical minimum and maximum values to the Qiagen method, the MR values were not normally distributed, showing ‘artificial’ skewing toward the hypermethylated state. Based on this result, as well as on the throughput, time for manual sample handling requirements and cost, the automated bisulfite conversion method utilizing

© 2022 Godler DE et al. *JAMA Network Open.*

Qiacube HT (Qiagen, Hilden, Germany), was chosen for the large-scale prevalence and feasibility studies.

eAppendix 7: Validation of Competitive Priming Initiated Nested (CINQ) ddPCR

The quantitative methylation analysis was validated on PWS and AS DNA spiked at different ratios, with expected versus observed methylation ratio showing high degree of correlation. When applied to 44 controls and 57 DNA samples from individuals affected with chromosome 15 imprinting disorders, CINQ ddPCR showed sensitivity as specificity of 100%, for PWS, AS and Dup15q. The UPD subtype of PWS showed greater variation than the deletion and ICD subtypes, suggesting presence of mosaicism specific to UPD, that may not have been identified as part of diagnostic testing, due to potential differences in analytic sensitivity.

CINQ ddPCR also showed sensitivity and specificity of 100% to differentiate blood spots from 58 individuals affected with chromosome 15 imprinting disorders from 44 control NBS from the general population (with *SNRPN* methylation ratio of 0.5 by MS-QMA). Moreover, CINQ ddPCR showed less variation in the normal range (0.59 to 0.72 MR) from controls with high quality DNA extracted in diagnostic settings, as compared to crude DNA extracts from archival NBS samples (normal range 0.52 to 0.74 MR).

© 2022 Godler DE et al. *JAMA Network Open*.

eTable 1. Positive predictive values (PPV) and prevalence estimates from 16,579 NBS fanalysed with MS-QMA.

	AS	PWS	Dup15q	All C15 Disorders
**Positive threshold based on population mean +/- 3 (STDEV)**	<0.256	>0.768	Mat. >0.768<br>Pat. <0.256	>0.768<br><0.256
**Positive MS-QMA Result No**	19	73	92	92
**Confirmed Positive Result No**	2	2	1	5
**Positive Predictive Value (PPV)**	**10.5%**	**2.7%**	**1.1%**	**5.4%**
**Prevalence**	1:8,290	1:8,290	1:16,579	1:3,316
**95% confidence interval**	(1:2,297-1:68,493)	(1:2,297-1:68,493)	(1:2,978-1:653,595)	(1:1422-1:10,214)
**Positive threshold Based on PWS and AS DBS from diagnostic settings***	≤ 0.12	≥ 0.88	N/A	≤ 0.12<br>≥ 0.88
**Positive MS-QMA Result No**	3	6	N/A	9
**Confirmed Positive Result No**	2	2	N/A	4
**Positive Predictive Value (PPV) %**	**67%**	**33%**	N/A	**44%**
**Prevalence**	1:8,290	1:8,290	N/A	1:4,145
**95% confidence interval**	(1:2,297-1:68,493)	(1:2,297-1:68,493)	N/A	(1:1,620-1:15,221)

Note: *Matrices in cells highlighted in grey utilize the positive threshold based on PWS and AS DBS from diagnostic settings. N/A: not applicable as Dup15 methylation range from DBS of cases confirmed to have this diagnosis in diagnostic settings overlaps with NBS control range. STDEV = standard deviaitons.

© 2022 Godler DE et al. *JAMA Network Open.*

eTable 2. Demographic information and *SNRPN* methylation ratio (MR) comparisons of the samples from the validation cohort.

	**Controls**		**PWS**		**AS**		**matDup15q**		**T15/UPD**		**Controls v. PWS**	**Controls v. AS**	**PWS v AS**
	**n**	**%**	**n**	**%**	**n**	**%**	**N**	**%**	**n**	**%**	**P**	**p**	**p**
**Male (%)**[a]	37	35.1	72	50.0	44	45.5	9	66.7	2	100	.159	.373	.703
	**n**	**Md (IQR)**	**n**	**Md (IQR)**	**n**	**Md (IQR)**	**N**	**Md (IQR)**	**n**	**Range**			
**Age**[b]	26	42.00 (15.50)	69	3.00 (17.92)	43	2.76 (10.62)	9	4.00 (6.00)	2	0.50-3.75	**<.001**	**<.001**	.433
***SNRPN* (MR)**	37	0.531 (0.042)	75	0.994 (0.005)	46	0.025 (0.010)	9	0.745 (0.054)	2	0.896-0.931	**<.001**	**<.001**	**<.001**

Note. Fisher's Exact test compared proportion of males (%) between groups. Mann Whitney U test was used to compare groups on age and *SNRPN* (MR). Group comparisons for matDup15q and T15/UPD were not completed due to small sample sizes.
[a]3 PWS and 2 AS did not have sex reported.
[b]11 controls, 6 PWS and 3 AS did not have age reported.
AS = Angelman syndrome; matDup15q = maternal chromosome 15q duplication syndrome; Md = Median; IQR = Interquartile range; MR = methylation ratio; PWS = Prader-Willi syndrome; T15/UPD = PWS with Trisomy 15/Uniparental Disomy.

© 2022 Godler DE et al. *JAMA Network Open.*

eTable 3. Demographic information and *SNRPN* methylation ratio (MR) for confirmatory testing of PWS and AS samples stratified by tissue type from the validation cohort.

	Sex		Age		*SNRPN* (MR)	
	**n**	**% male**	**n**	**Md (IQR)**	**N**	**Md (IQR)**
**PWS**						
NBS	10	60.0	10	5.02 (21.02)	10	0.987 (0.022)
DBS	12	58.3	12	8.08 (20.12)	12	0.995 (0.013)
Blood	58	41.4	54	1.88 (7.38)	58	0.995 (0.005)
Buccal/Saliva[a]	5	100.0	3	24.17-30.00	5	0.991 (.006)
**AS**						
NBS[b]	3	100.00	3	2.76-24.48	3	0.033-0.502
DBS	6	50.0	6	5.79 (4.59)	6	0.045 (0.503)
Blood	39	41.0	36	2.25 (10.88)	39	0.020 (0.010)

Note. Controls, matDup15q and PWS with Trisomy 15/Uniparental Disomy (T15/UPD) groups only provided blood samples. Demographic information for these groups is included in eTable 1.

[a]Range provided for age as n=3

[b]Range provided for age and *SNRPN* (MR) as n=3

AS = Angelman syndrome; DBS = dried bloodspot; IQR = Interquartile range; NBS = Newborn bloodspot; Md= Median; MR = methylation ratio.

© 2022 Godler DE et al. *JAMA Network Open*.

eTable 4. Distribution of *SNRPN* Methylation Ratio (MR) and comparisons between sexes for the NBS samples from the general population from the MS-QMA validation cohort.

	Whole cohort		Male		Female		Unknown sex		Males v Females
	**N**	**M ± SD**	**n**	**M ± SD**	**n**	**M ± SD**	**n**	**M ± SD**	**p**
**Qiagen**									
***SNRPN* (MR)**	831	0.526 ± 0.058	396	0.524 ± 0.061	364	0.525 ± 0.054	71	0.534 ± 0.064	.813
**Zymo**									
***SNRPN* (MR)**	322	0.576 ± 0.074	162	0.576 ± 0.074	160	0.576 ± 0.075	-	-	.926

Note. Independent samples t-test compared males and females on *SNRPN* (MR)
Md = Median; IQR = Interquartile range; MR = methylation ratio.

© 2022 Godler DE et al. *JAMA Network Open*.

eTable 5. Distribution of *SNRPN* Methylation Ratio (MR) and comparisons between sexes for the NBS samples from the general population from the MS-QMA test cohort.

	**Whole cohort**		**Male**		**Female**		**Unknown sex**		**Males v Females**
	**n**	**M ± SD**	**n**	**M ± SD**	**n**	**M ± SD**	**n**	**M ± SD**	**p**
***SNRPN* (MR)**	16579	0.512 ± 0.085	8086	0.512 ± 0.087	8153	0.512 ± 0.084	340	0.514 ± 0.080	.544

Note. Independent samples t-test compared males and females on *SNRPN* (MR)
Md = Median; IQR = Interquartile range; MR = methylation ratio.

© 2022 Godler DE et al. *JAMA Network Open*.

eTable 6. Summary of the numbers and statistics for the samples analyzed for *SNRPN* methylation levels using MS-QMA in newborn blood spots (NBS) from 16,579 infants. Note: * Ranges of methylation ratio values expected for different chromosome 15 imprinting disorders were established on diagnostic samples. STDEV = standard deviation.

**Statistic**	**Methylation Ratio**	**Methylation Ratio Range**	**Total Number: 92**
		AS: 0-0.1	2
Mean	0.51	Mosaic AS or pat Dup15q: 0.11 – 0.19	1
Median	0.50	Mosaic AS or pat Dup15q: 0.20 - 0.256	16
STDEV	8.5	PWS: 0.90 – 1.00	6
Mean + 2 STDEV	0.68	Mosaic PWS or mat Dup15q: 0.8 – 0.899	31
Mean - 2 STDEV	0.34	mat Dup15q: 0.78 – 0.799	20
		mat Dup15q: 0.768 – 0.779	16

© 2022 Godler DE et al. *JAMA Network Open.*

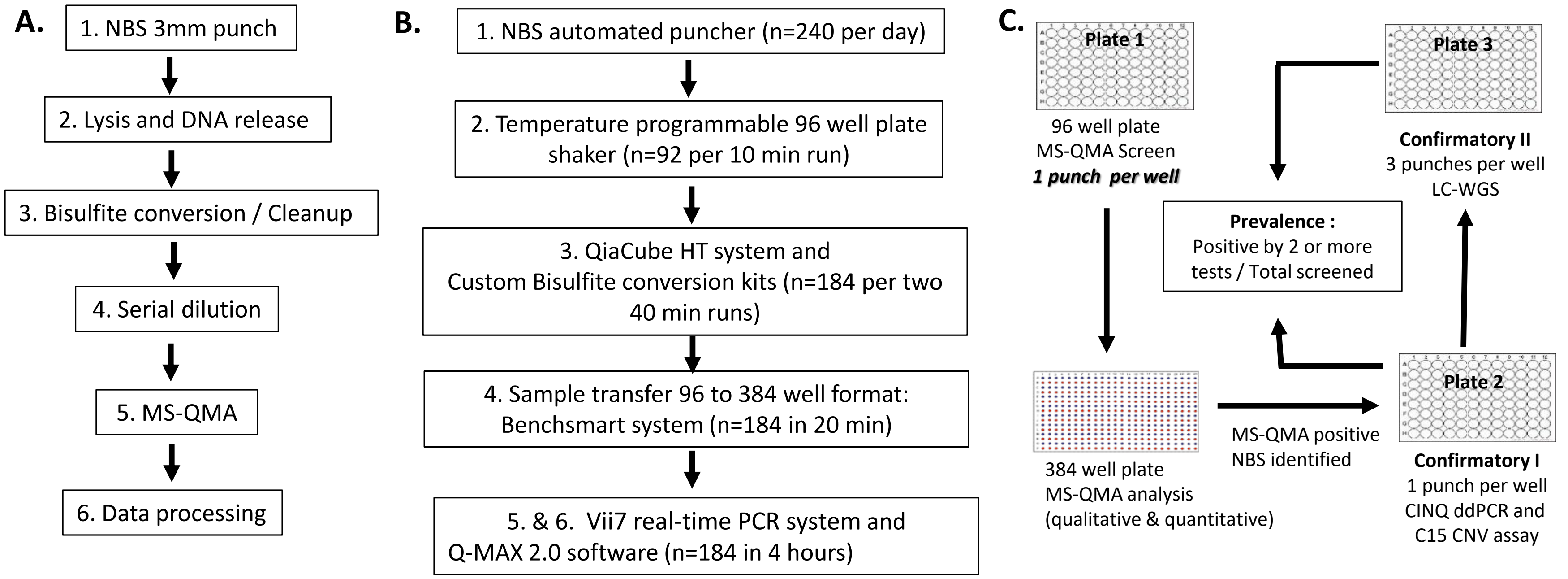

eFigure 1. Screening and confirmatory testing workflow developed for prevalence studies on newborn blood spots consented for de-identified research. (A) Steps involved in 1st-tier screening using MS-QMA analysis of *SNRPN* methylation on a single 3mm newborn blood spot (NBS) per infant performed in 96 well format. **(B)** Equipment / software required and throughput per day. Note: Numbers in **(B)** correspond to step numbers in **(A)**, with n indicating the number of NBS samples. **(C)** Every set of 93 NBS cards were punched into 3 separate replicate barcoded plates with the same 3 positive and negative DBS controls included on each plate. Plate 1 and 2 had a single 3mm punch per well, while plate 3 had three 3mm punches per well. Plate 1 was used for 1st-tier screening, while plates 2 and 3 were used for confirmatory testing using CINQ ddPCR analysis of *SNRPN* methylation; PWS/AS Imprinting centre copy number variation (CNV) analysis using real-time PCR; and chromosome 15 analysis using LC-WGS.

© 2022 Godler DE et al. *JAMA Network Open.*

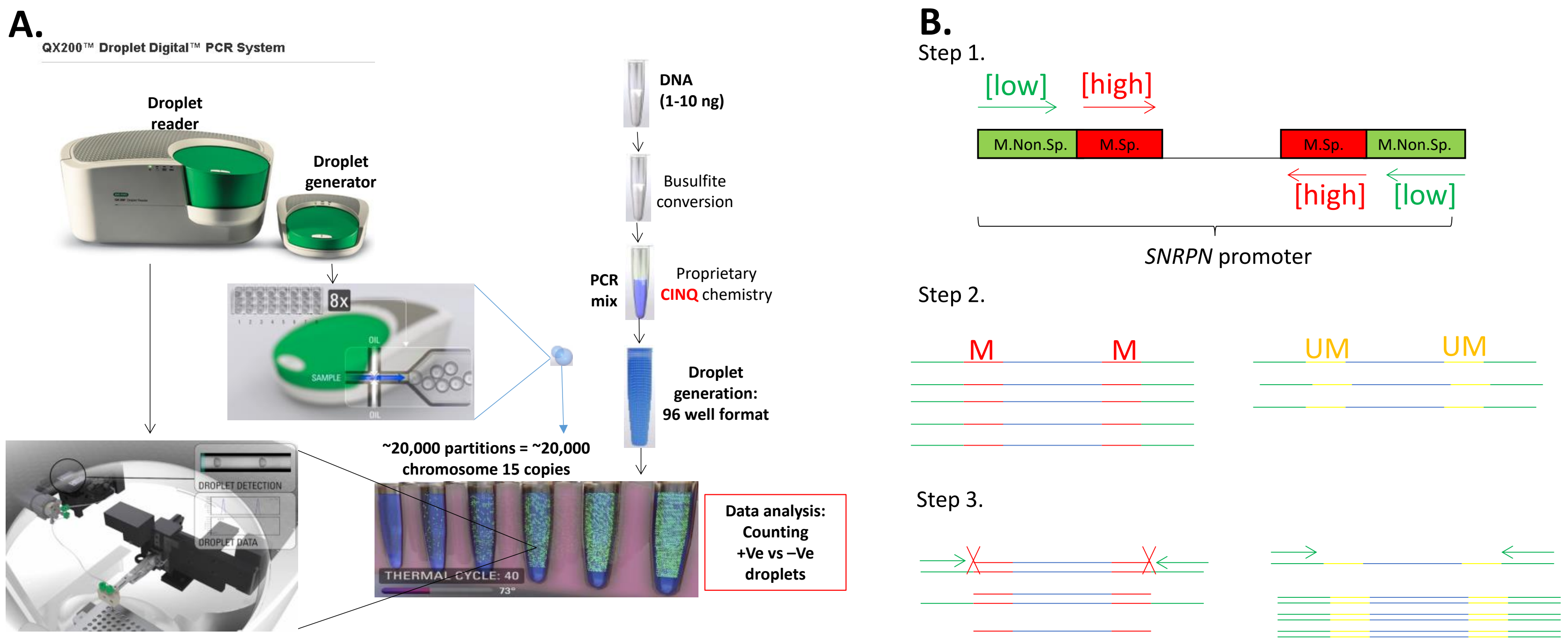

eFigure 2. Competitive Priming Initiated Nested Quantification using droplet digital PCR (CINQ ddPCR). (A) CINQ ddPCR analysis is performed using total of 1 to 10 ng DNA per sample which is initially bisulfite converted, and then amplified using CINQ chemistry that contains primers that are either specific for only amplicons from methylated DNA sequences; or that are non-specific for DNA methylation, and a dye that binds double-stranded DNA. Following, PCR, QX200 system was used to generate 20,000 droplets per sample that either contained amplicons from methylated or unmethylated target sequences. The system than counted the droplets that had different fluorescence amplitude. This amplitude was used to differentiate the methylation status of droplets that either contained amplicons from methylated or unmethylated *SNRPN* sequences. The methylation ratio was then calculated for each sample representing total number of positive droplets with methylation positive amplicons / total number of positive droplets. **(B)** CINQ ddPCR chemistry involved internal primers (in red) preferentially amplifying the target product due to higher concentration than external primers that amplified methylated and unmethylated sequences with equal efficiency. This difference in concentration as well as location of the primers transformed methylation non-specific primers (in green) into becoming specific for the flanking unmethylated sequences. Because there was a difference in size of amplicons from methylated and unmethylated sequences their droplets had a different amplitude. This difference was used to define droplet populations generated by this method.

© 2022 Godler DE et al. *JAMA Network Open*.

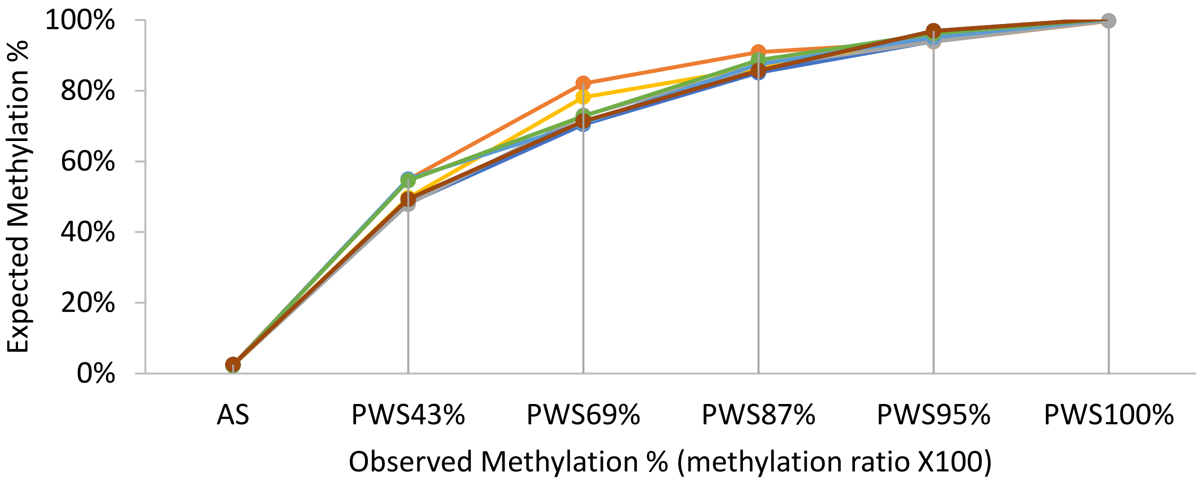

Expected Meth. %	RUN1	RUN2	RUN3	RUN4	RUN5	RUN6	RUN7	RUN8	Mean	2STDEV
0%	2%	2%	2%	2%	2%	2%	2%	2%	2%	**2%**
43%	48%	54%	55%	48%	50%	55%	55%	49%	51%	**8%**
69%	70%	76%	82%	73%	78%	71%	73%	71%	74%	**8%**
87%	85%	86%	91%	87%	87%	87%	89%	86%	87%	**4%**
95%	94%	98%	94%	94%	96%	95%	96%	97%	95%	**3%**
100%	100%	101%	100%	100%	100%	100%	100%	100%	100%	**1%**

eFigure 3. Assessing variability in *SNRPN* methylation output between 8 runs using the same control spiked lymphoblast DNA samples on each plate. Note: PWS female DNA (100% methylated *SNRPN*) and AS male DNA (0% methylated *SNRPN*) were mixed at different ratios. The plot indicates relationship between observed and expected methylation percentage. The table indicates variability for each spiked sample between 8 runs / plates represented by 2 standard deviation (2STDEV).

© 2022 Godler DE et al. *JAMA Network Open*.

A.

		PWS as predicted condition (≥0.88 MR cut-off)		
	Total number of samples: 1,356	**Positive**	**Negative**	
**PWS as actual condition**	Positive: 109	TP: 108	FN: 1	Sensitivity: 99%
	Negative: 1,247	FP: 0	TN: 1,246	Specificity: 100%
		PPV: 100%	NPV: 99.9%	

B.

		AS as predicted condition (≤0.12 MR cut-off)		
	Total number of samples: 1,356	**Positive**	**Negative**	
**AS as actual condition**	Positive: 48	TP: 45	FN: 3	Sensitivity: 93.8%
	Negative: 1,308	FP: 0	TN: 1,306	Specificity: 100%
		PPV: 100%	NPV: 99.9%	

C.

		Dup15q as predicted condition (>0.7 or <0.33 MR cut-off)		
	Total number of samples: 1,356	**Positive**	**Negative**	
**Dup15q as actual condition**	Positive: 9	TP: 7	FN: 2	Sensitivity: 77.8%
	Negative: 1,347	FP: 1	TN: 1,346	Specificity: 99.9%
		PPV: 87.5%	NPV: 99.8%	

eFigure 4. Sensitivity, specificity, positive (PPV) and negative predictive value (NVP) calculations for *SNRPN* methylation analysis using Methylation Specific Quantitative Melt Analysis (MS-QMA) from Figure 1 to differentiate: (A) PWS, (B) AS and (C) Dup15q from other samples. Note: TP = true positive; FP = false positive; FN = false negative; TN = true negative. PPV % = (TP/TP +FP) X 100; NVP % = TN/TN + FN) X 100; Sensitivity = (TP/[TP+FN]) X 100; Specificity = (TN/TN+FP)X100.

© 2022 Godler DE et al. *JAMA Network Open*.

Figure S4

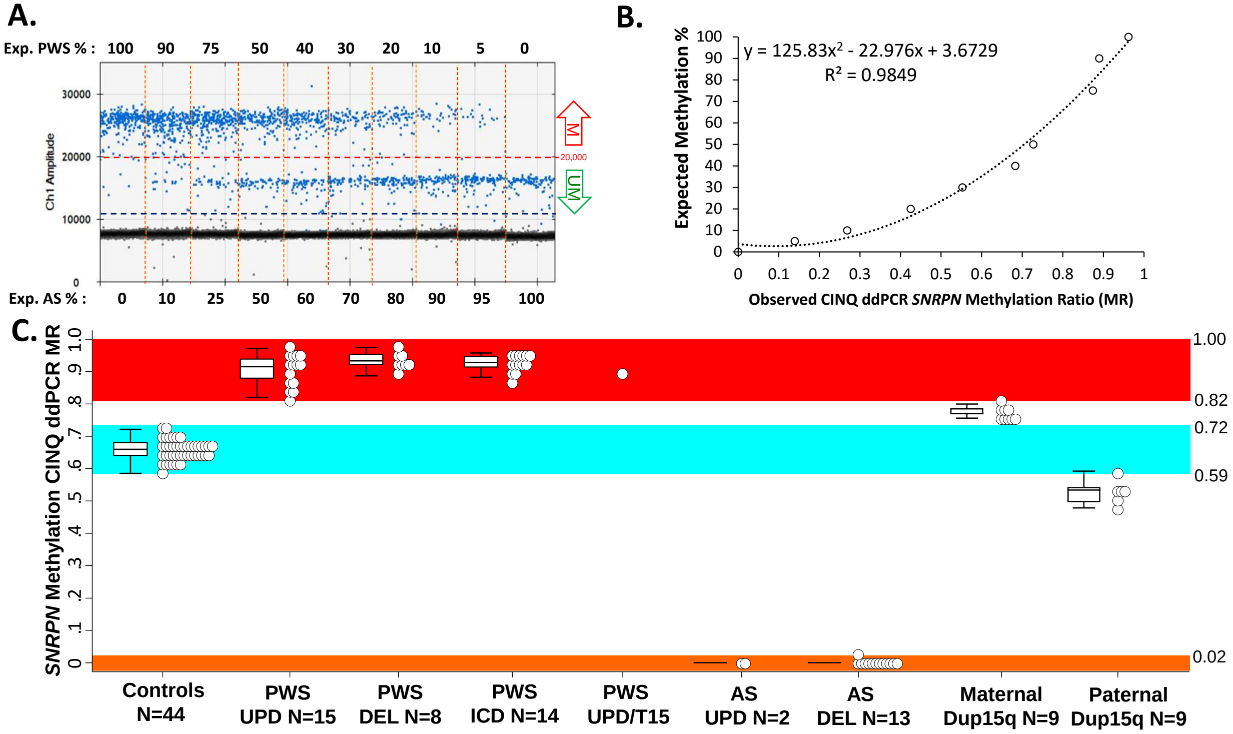

eFigure 5. CINQ droplet digital PCR validation for quantitative analysis of *SNRPN* methylation. (A) 2-D plot from a spiking experiment combining PWS and AS DNA from lymphoblast cell lines at different ratios, indicated as different percentages above and below each plot (X-axis). Each column separated by broken red lines represents a result for a separate sample. Each blue dot represents a single positive copy of *SNRPN* promoter either maternally inherited and methylated (red arrow) or paternally inherited and unmethylated (green arrow). The amplitude (Y-axis) represents the difference in fragment size amplified by the assay. **(B)** Scatter plot of the relationship between observed methylation ratio (MR) and expected methylaiton p[ercentage of AS DNA combined with PWS DNA, at different percentages (Y-axis). The X-axis represents the ratio between the total number of positive droplet of maternally inherited methylated alleles divided by the total positive droplets from maternally and paternally inherited alleles. Line of best fit, the equation and the coefficient of correlation (R^2) for the relationship are also included. **(C)** *SNRPN* methylation ratio from CINQ ddPCR analysis on blood and saliva DNA including: 44 typically developing controls and diagnostic samples from 24 PWS, 1 PWS mosaic for UPD and trisomy 15 (UPD/T15), 15 AS and 18 Dup15q affected individuals. Note: Molecular diagnosis for these samples was confirmed using chromosomal microarray, Methylation-Specific Multiplex Ligation-Dependent Probe Amplification or methylation sensitive PCR targeting *SNRPN* promoter methylation.

© 2022 Godler DE et al. *JAMA Network Open.*

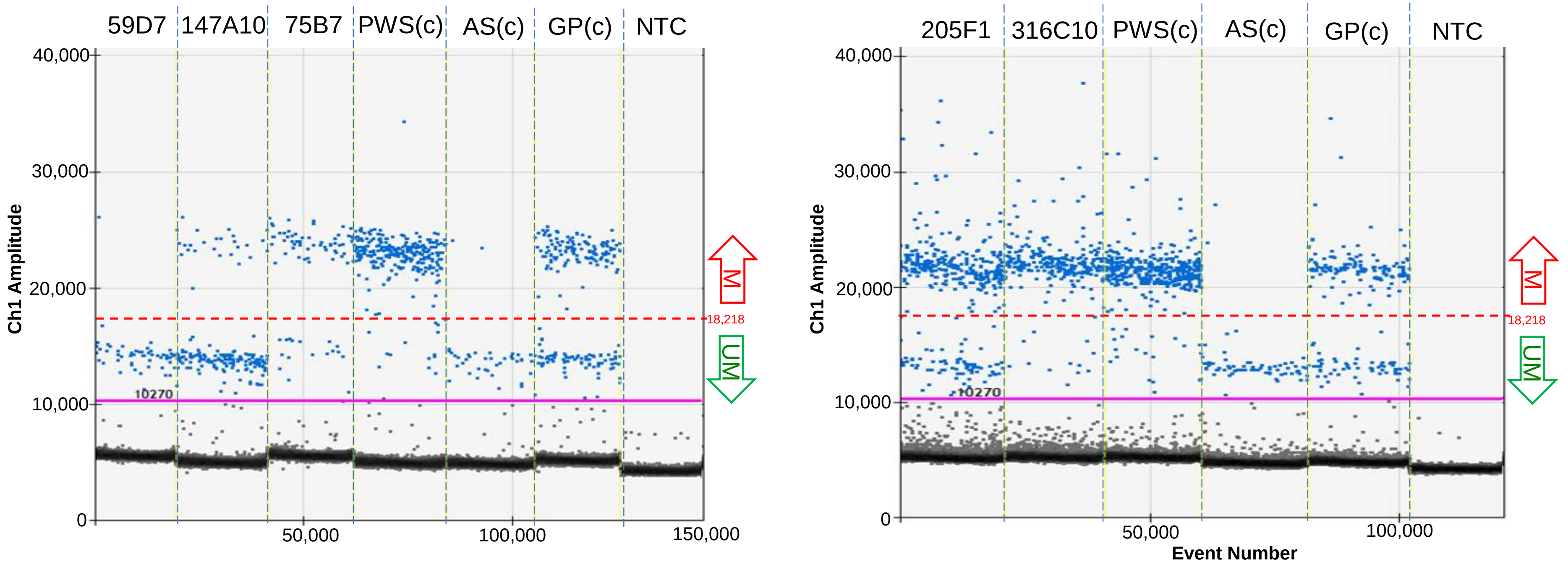

eFigure 6. Examples of 2-D plots from CINQ ddPCR for NBS confirmed to have abnormal *SNRPN* methylation co-run with DBS from positive and negative controls. Note: each blue dot indicated data from a single ddPCR droplet; each column contains data for a single sample; purple line indicates amplitude cut-off above which all droplets were considered to have positive signal above background; red horizontal line is the amplitude cut-off above which all droplet signals were considered to be from maternal alleles with methylated *SNRPN*; droplet with amplitude between purple and red line cut-offs were considered to be from paternal alleles with unmethylated *SNRPN;* red M = methylated allele signal; green UM = unmethylated allele signal; PWS(c) = PWS positive control DBS; AS(c) = AS positive control DBS; GP(c) = general population infant DBS negative control; NTC = no template control.

© 2022 Godler DE et al. *JAMA Network Open.*

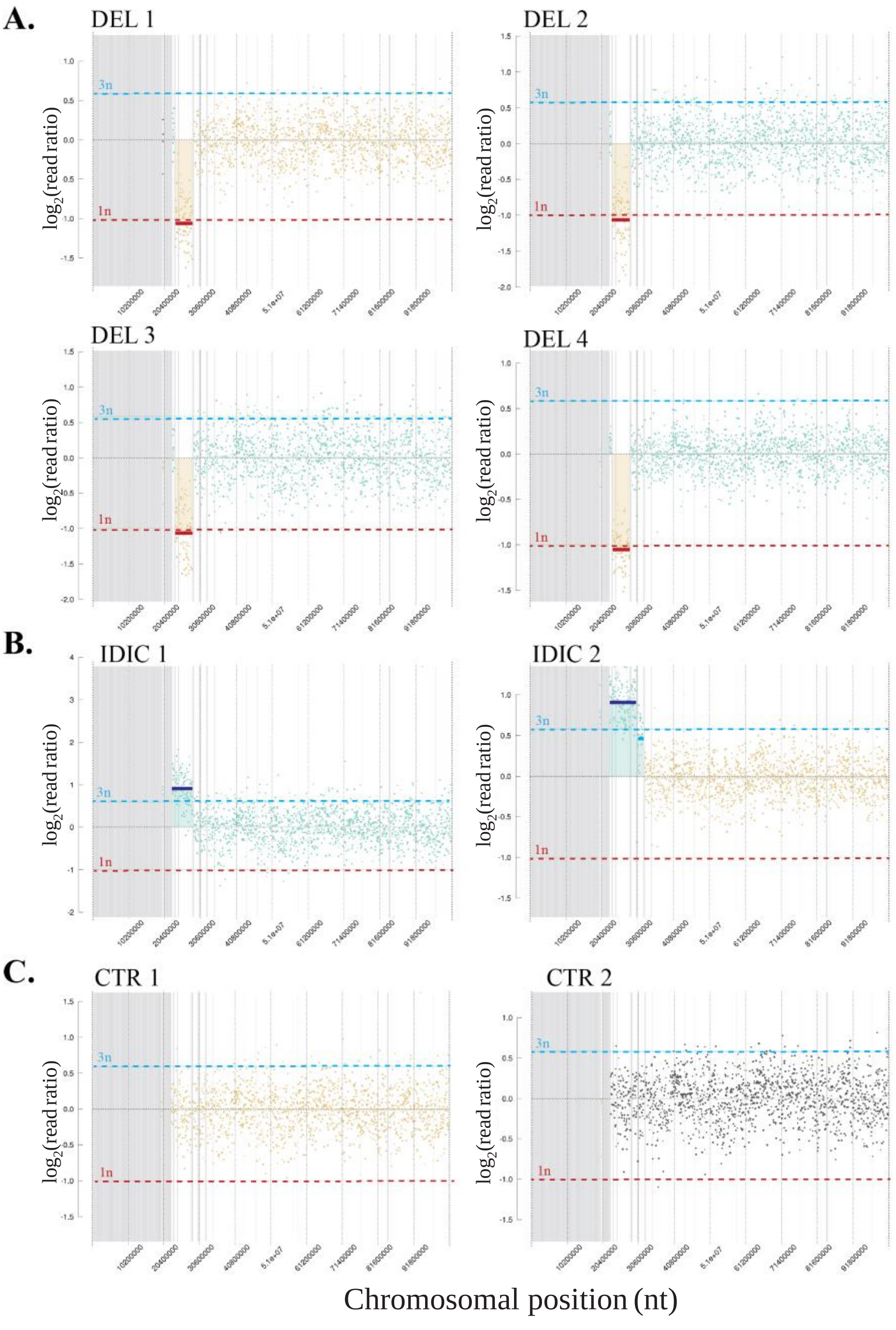

eFigure 7. Validation of confirmatory testing using Low-Coverage Whole Genome Sequencing (LC-WGS). LC-WGS analysis of DBS samples from 6 individuals with confirmed diagnosis through standard diagnostic testing: **(A)** deletion of the 15q11-3 classical PWS/AS imprinted region (DEL); (B) isodicentric 15q (IDIC), and **(C)** typically developing controls (CTR). The method differentiated between 4 deletion cases, 2 IDIC cases and 2 controls. Note: blue broken line represents threshold for the relative number of 3 copies, while red broken line represents the threshold for the relative number of 1 copy. The grey line with the $\log_2$(ratio) of 0 (y-axis) represents the threshold for the relative number of 2 copies.

© 2022 Godler DE et al. *JAMA Network Open.*

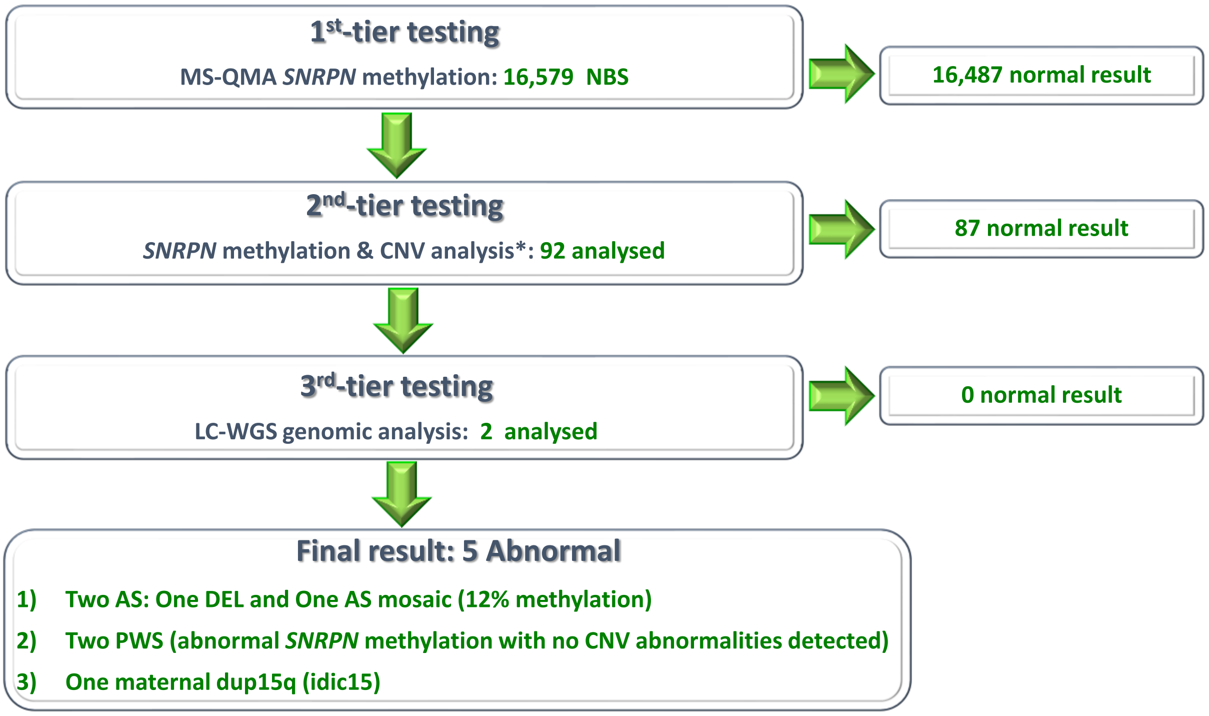

eFigure 8. Summary of newborn blood spots tested, and the positive cases identified by 1st, 2nd and 3rd-tier testing. Note: *For second-tier testing 69 NBS had both CINQ ddpCR and CNV analysis performed; 23 only had methylation analysis performed as there was insufficient sample remaining for the CNV analysis.

© 2022 Godler DE et al. *JAMA Network Open*.

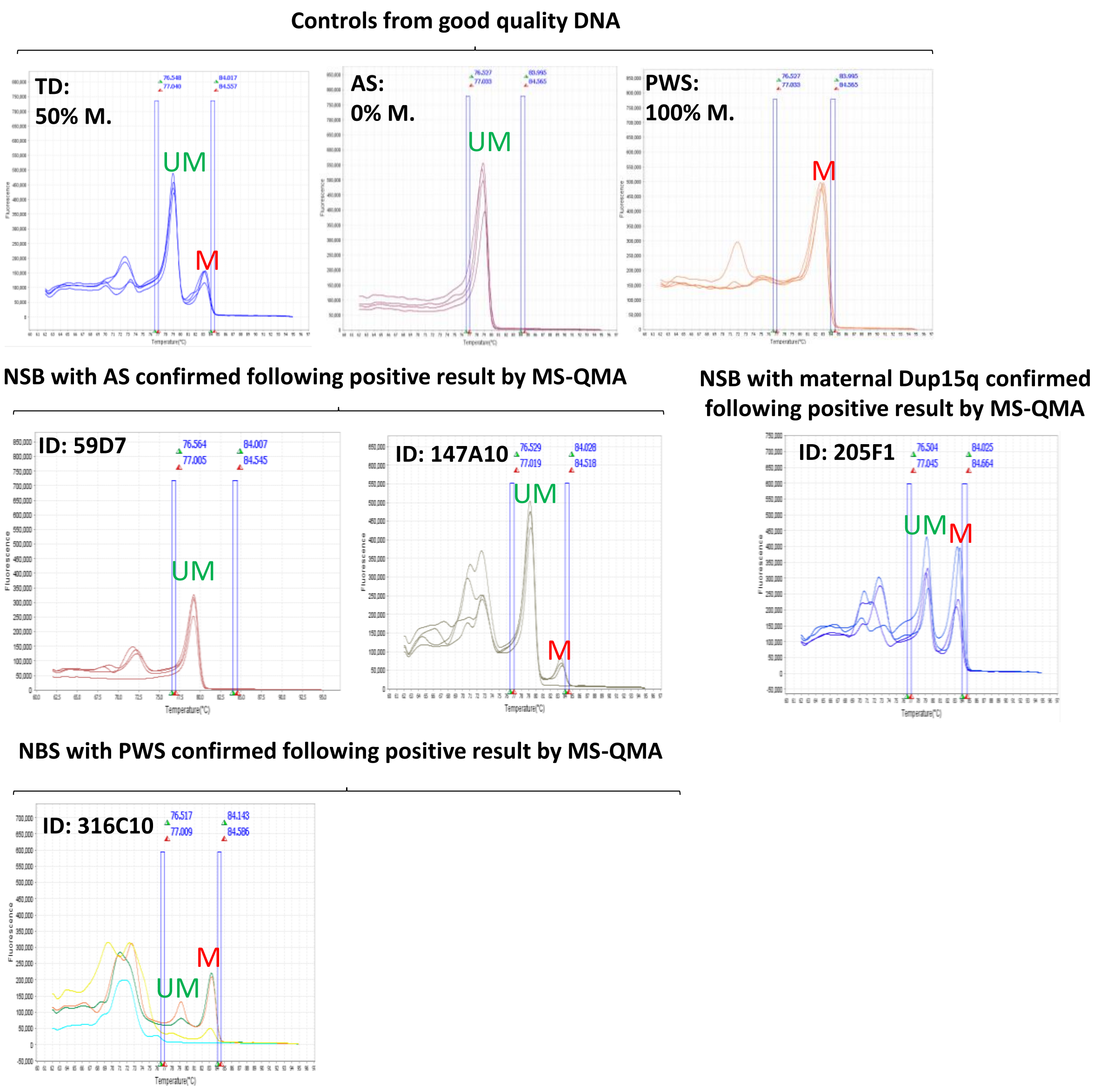

eFigure 8. Derivative curve profiles of 5 cases positive by quantitative methylation analysis using MS-QMA. Note: UM – an unmethylated allele peak; M – a methylated allele peak. For each plot X-axis indicates high resolution melt (HRM) temperature, while Y-axis indicates fluorescence units of double stranded DNA intercalating dye released as part of HRM. Note: TD – neuro-typical control; PWS – Prader Willi Syndrome; AS - Angelman Syndrome.

© 2022 Godler DE et al. *JAMA Network Open*.
